# Supplementary material for: Generating colorblind-friendly scatter plots for single-cell data
Source: eLife. 2022 Dec 16;11:e82128. doi: 10.7554/eLife.82128 (PMC9829408; doi:10.7554/eLife.82128)
Supplement: Supplementary file 1. [file elife-82128-supp1.pdf]

Creating a Scatterplot with Texture

Tejas Guha

November 5, 2022

Installing ScatterHatch

scatterHatch requires R (version 4.2) and can be installed through BioConductor using the following commands.

```
if (!require("BiocManager", quietly = TRUE))
    install.packages("BiocManager")

BiocManager::install("scatterHatch")
```

If you do not have BiocManager, the installation can be done using the *devtools* package.

```
library(devtools)
install_github("FertigLab/scatterHatch")
```

Once scatterHatch has been successfully installed run the following command to load *scatterHatch* onto your R session.

```
library(scatterHatch)
```

Preparing the Data

The data that will be used to showcase the function is a tissue-CyCIF PDAC dataset from Lin et al. The preprocessing begins by adding manual annotations of each cell's location in the tissue sample.

```
data("pdacData")
pdacData$cellID = paste0('cell_', 1:nrow(pdacData))
pdacData$Yt <- -pdacData$Yt
pancreas_frames = c(1:6, 27:31, 15:19, 40:44)
PDAC_frames = c(23:26, 35:37, 51:52, 64:65, 77)
small_intestines_frames = c(49:50, 63, 75:76, 88:89, 100:103, 112:116, 125:129, 137:140)
annotateLocation <- function(frame){
    if (frame %in% pancreas_frames){return("Pancreas")}
    if (frame %in% PDAC_frames){return("PDAC")}
    if (frame %in% small_intestines_frames){return("Small Intestine")}
    return("Other")
}
pdacData$location = sapply(pdacData$frame, annotateLocation)

head(pdacData[, c('Xt', 'Yt', 'location', 'frame')])
#>         Xt        Yt location frame
#> 1 1342.878  -801.154 Pancreas     1
#> 2 5688.494 -1391.393 Pancreas     4
#> 3 6295.826 -1393.807 Pancreas     4
#> 4 5344.257 -1391.650 Pancreas     4
#> 5 5640.034 -1391.416 Pancreas     4
#> 6 5923.357 -1390.776 Pancreas     4
```

Creating a Basic ScatterHatch Plot

`scatterHatch()` must have a data frame passed to it, along with three strings denoting the columns with the x/y coordinates and a factor variable of each point being plotted. The factor variable identifies the group each point is a part of. `scatterHatch()` returns a ggplot2 object with three layers; the points, the line segments (the hatching), and an invisible custom geom to render the legend icons.

```
plt <- scatterHatch(data = pdacData, x = "Xt", y = "Yt",
    color_by = "location", legendTitle = "Tissue Type")
plot(plt)
```

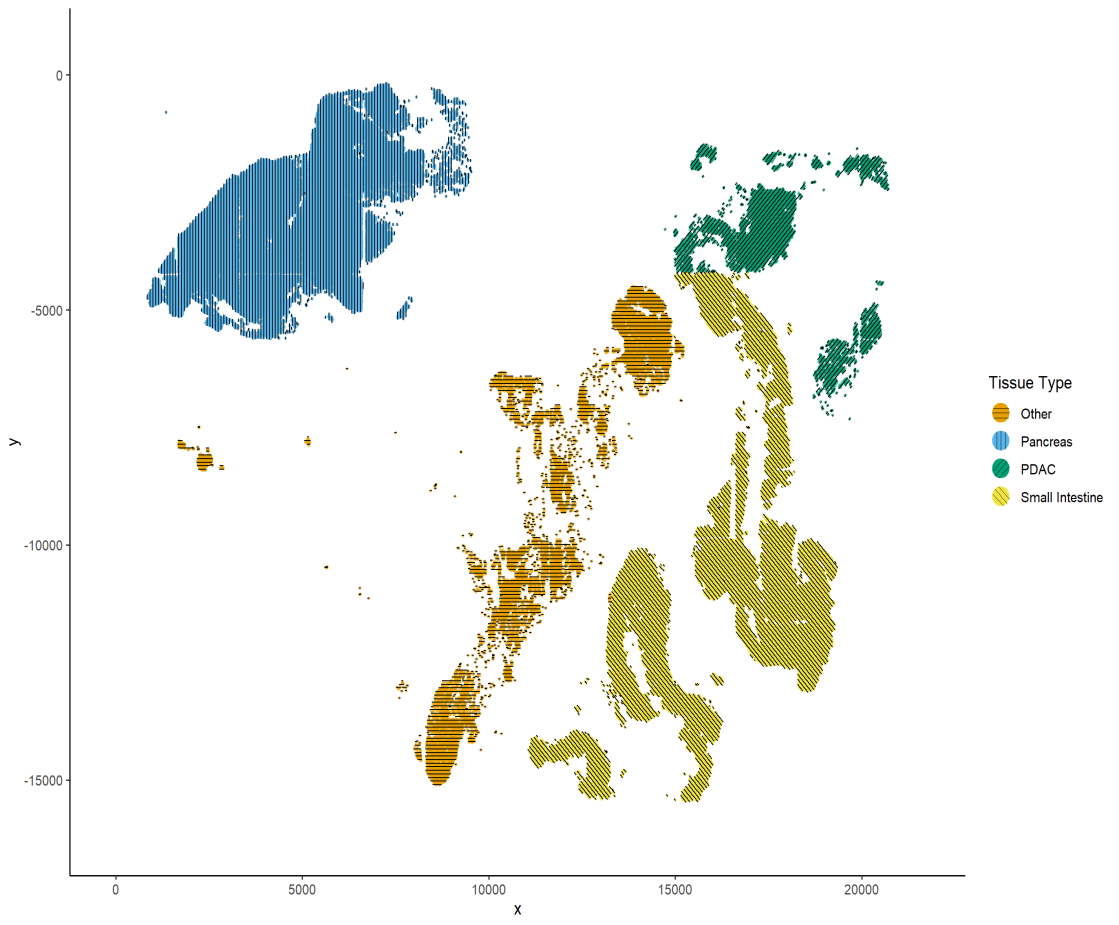

Customizing ScatterHatch Plot

Changing the Order of Pattern Assignment

Controlling the aesthetics of each pattern is achieved by passing a list to the `patternList` argument. Each element of the list should contain a list that denotes the desired aesthetics for each pattern. Every element of `patternList` must have a named element called "pattern" that describes the unique pattern type. If patternList is passed, then the length of patternList must be equal to the number of groups being plotted.

Changing the Angles of each Pattern

Angle denotes the angle(s) for which the lines of a particular hatching pattern should be drawn. For example, the default horizontal pattern ("-") is an angle of 0 while the default vertical pattern ("|") is an angle of 90. Angle can be a vector with multiple angles. For example, the cross ("x") is a vector of angles: `c(135, 45)`.

```
patternList = list(list(pattern="/", angle = 70), list(pattern="-"), list(pattern="x", angle = c(135,
         90, 45), lineWidth = 0.2), list(pattern="+"))
plt <- scatterHatch(data = pdacData, x = "Xt", y = "Yt",
    color_by = "location", legendTitle = "Tissue Type",
    patternList = patternList)
plot(plt)
```

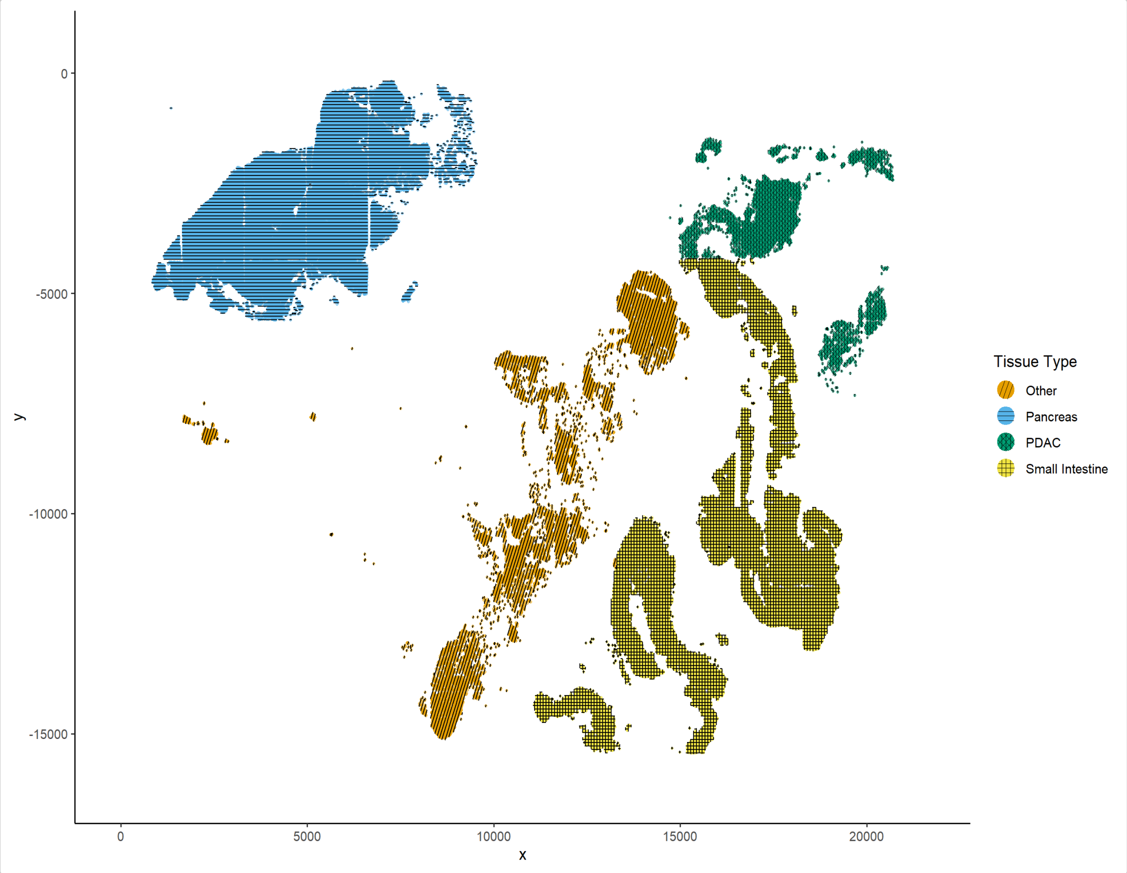

scatterHatch() Arguments Explained

Argument	Description
data	A dataframe of the dataset being plotted
x	A string describing the column name with the x-coordinates of the points being plotted
y	A string describing the column name with the y-coordinates of the points being plotted
factor	A string describing the column name of the factor variable
legendTitle	The legend title
pointSize	ggplot2 point size
pointAlpha	Transparency of each point
gridSize	Integer describing the precision of the hatched patterns. Larger the value, greater the precision at the expense of efficiency. Default segregates plots into 10000 bins
sparsePoints	A logical vector that denotes the outlying points. Default utilizies an in-built sparsity detector
patternList	List containing the aesthethics of each pattern
colorPalette	Character vector describing the point color of each group; default is color-blind friendly and uses colors from the dittoSeq package

Pattern Aesthetics Arguments

These pattern aesthetics are passed into a list in the patternList argument of `scatterHatch()`.

Aesthetics	Description
pattern	A string representing one of the possible 7 patterns to be used: (horizontal or '-'), (vertical or '|'), (positiveDiagonal or '/'), (negativeDiagonal or '\\'), (checkers or '+'), (cross or 'x'), and (blank or '').
angle	Vector or number denoting at what angle(s) the lines in a hatching pattern should be drawn - e.g. `c(45, 90, 135)`
lineWidth	Number representing the width of the lines in the pattern
lineColor	String representing the colors of the lines in the pattern (black, white, etc.)

Aesthetics	Description
lineType	String representing the type of lines in the pattern (solid, dashed, etc.)

Session Info

```
sessionInfo()
#> R version 4.0.2 (2020-06-22)
#> Platform: x86_64-w64-mingw32/x64 (64-bit)
#> Running under: Windows 10 x64 (build 19044)
#>
#> Matrix products: default
#>
#> locale:
#> [1] LC_COLLATE=English_United States.1252
#> [2] LC_CTYPE=English_United States.1252
#> [3] LC_MONETARY=English_United States.1252
#> [4] LC_NUMERIC=C
#> [5] LC_TIME=English_United States.1252
#>
#> attached base packages:
#> [1] stats     graphics  grDevices utils     datasets  methods   base
#>
#> other attached packages:
#> [1] scatterHatch_0.99.2
#>
#> loaded via a namespace (and not attached):
#>  [1] Rcpp_1.0.8          highr_0.9           plyr_1.8.6
#>  [4] compiler_4.0.2      pillar_1.7.0        tools_4.0.2
#>  [7] digest_0.6.29       evaluate_0.15       lifecycle_1.0.1
#> [10] tibble_3.1.6        gtable_0.3.0        lattice_0.20-41
#> [13] pkgconfig_2.0.3     rlang_1.0.1         Matrix_1.2-18
#> [16] cli_3.2.0           rstudioapi_0.13     yaml_2.3.5
#> [19] xfun_0.29           fastmap_1.1.0       spatstat.data_2.2-0
#> [22] dplyr_1.0.8         stringr_1.4.0       knitr_1.37
#> [25] generics_0.1.2      vctrs_0.3.8         spatstat.utils_2.3-1
#> [28] tidyselect_1.1.2    grid_4.0.2          glue_1.6.2
#> [31] R6_2.5.1            fansi_1.0.2         spatstat.geom_2.4-0
#> [34] rmarkdown_2.11      polyclip_1.10-0     farver_2.1.1
#> [37] purrr_0.3.4         ggplot2_3.3.6       deldir_1.0-6
#> [40] magrittr_2.0.2      scales_1.2.0        htmltools_0.5.2
#> [43] ellipsis_0.3.2      colorspace_2.0-3    labeling_0.4.2
#> [46] utf8_1.2.2          stringi_1.7.6       munsell_0.5.0
#> [49] crayon_1.5.1
```
